# Eligibility of a novel BW + technology and comparison of sensitivity and specificity of different imaging methods for radiological caries detection

**DOI:** 10.1007/s11282-024-00748-4

**Published:** 2024-04-29

**Authors:** Kathrin Becker, Henrike Ehrlich, Mira Hüfner, Nicole Rauch, Caroline Busch, Beryl Schwarz-Herzke, Dieter Drescher, Jürgen Becker

**Affiliations:** 1 Department of Orthodontics and Dentofacial Orthopaedics, Charité–Universitätsmedizin Berlin, Aßmannshauser Str. 4-6, 14197 Berlin, Germany; 2 Department of Orthodontics, Universitätsklinikum Düsseldorf, Düsseldorf, Germany; 3 Department of Oral Surgery, Universitätsklinikum Düsseldorf, Düsseldorf, Germany; 4 Institute for Anatomy II, University of Düsseldorf, Düsseldorf, Germany

**Keywords:** Dental radiography, Digital imaging, Dental caries, Cone Beam CT, 3D-Radiology, Effective dose

## Abstract

**Objectives:**

Bitewing radiography is considered to be of high diagnostic value in caries detection, but owing to projections, lesions may remain undetected. The novel bitewing plus (BW +) technology enables scrolling through radiographs in different directions and angles. The present study aimed at comparing BW + with other 2D and 3D imaging methods in terms of sensitivity, specificity, and user reliability.

**Materials and methods:**

Five human cadavers were used in this study. In three cadavers, natural teeth were transplanted post-mortem. BW + , two-dimensional (digital sensors, imaging plates, 2D and 3D bitewing radiographs) and 3D methods (high and low dose CBCT) were taken. Carious lesions were evaluated on 96 teeth at three positions (mesial, distal, and occlusal) and scored according to their level of demineralization by ten observers, resulting in 35,799 possible lesions across all observers and settings. For reference, µCT scans of all teeth were performed.

**Results:**

Overall, radiographic evaluations showed a high rate of false-negative diagnoses, with around 70% of lesions remaining undetected, especially enamel lesions. BW + showed the highest sensitivity for dentinal caries and had comparatively high specificity overall.

**Conclusions:**

Within the limits of the study, BW + showed great potential for added diagnostic value, especially for dentinal caries. However, the tradeoff of diagnostic benefit and radiation exposure must be considered according to each patient’s age and risk.

## Introduction

With an estimated affected population of over 3 billion people, of which 530 million are children, dental caries is one of the most common chronic diseases worldwide despite being generally preventable [1]. According to the Global Burden of Disease Study 2017, oral disorders, the majority of which being caries, are listed as the most common health condition across all sexes and ages. In the US, for instance, 9 out of 10 adults between the ages 20–64 show evidence of prior dental caries (such as the presence of dental restorations) and 27% have untreated tooth decay [2].

Accordingly, early and accurate detection of carious lesions is of high importance in dental practice. Visual and tactile examinations remain the standard method when first viewing a patient. However, radiographic imaging may be indicated and recommended for added diagnostic value, especially in high-risk patients. They are particularly useful for more advanced cases of fissure caries and interproximal caries. These lesions may not always be visible due to narrow interdental spaces, leading to false-negative results [3, 4].

Of the various imaging methods available, bitewing radiographs (first proposed by Raper et al. in 1925 [5]), are reported to have higher sensitivity than visual-tactile examination, and to be more sensitive than periapical or panoramic radiography for posterior interproximal caries detection [6–10].

While intraoral bitewing radiography is commonly employed, extraoral bitewing radiographs may be potentially preferable for patients reporting discomfort during intraoral bitewing imaging, especially with digital sensors, which tend to be thicker, and are commonly connected to a wire [11, 12].

Sirona Dental Systems GmbH (Bensheim, Germany) recently developed a novel method for extraoral bitewing radiography termed *Bitewing* + (BW +). These radiographs are produced through tomosynthesis, i.e. images are permanently recorded during the movement of the detector and the sensor, and thus images with multiple layers and different angles can be reconstructed, thus creating a 3D-like effect. For BW + diagnostics, this makes it possible to adjust angles and scroll through slices. However, this technique is available only with the 3D sensors, whereas for conventional extraoral bitewings, the conventional panoramic sensor (with decreased slit opening at the aperture) could be used. Therefore, a higher radiation dose is expected with BW + .

The aim of this study is to compare the sensitivity and specificity of BW + to conventional intra- and extraoral 2D bitewing radiographs as well as with 3D imaging using cone beam computed tomography (CBCT). In addition, the inter- and intrarater reliability were measured, and subjective perception of the first BW + usage was evaluated. Furthermore, the study aimed to assess the effective dose associated with BW + .

## Materials and methods

### Study participants

Ten clinicians of the Department for Oral Surgery of the University Hospital Düsseldorf, Germany, with expertise in dental radiology and cone beam computed tomography (CBCT), participated in the study. All observers evaluated the radiographs at the same time. The rating was performed in a single radiographic examining room with reduced ambient light. Each observer used a separate calibrated monitor.

## Specimen preparation

Ten human cadavers from the Institute for Anatomy II, University of Düsseldorf were provided following approval of the appropriate ethical committee (reference number: 2018-130-FmB). The cadavers had been freshly frozen at −20 °C and were, therefore, not embalmed. Prior to the radiographs, the specimens were slowly defrosted.

Two of the ten cadavers retained their natural dentition and the remaining eight were edentulous. Extracted teeth from hospital patients (collected in the clinic) were transplanted into the upper and lower jaws of three of the edentulous cadavers. The remaining cadavers were not used in this investigation. In two of three edentulous cadavers, two different dentitions were used. In total, 96 natural teeth were evaluated for every imaging technology in this study.

## Radiological imaging

Radiographs were made of the posterior regions of every dentition using eight two-dimensional (2D) and two–three-dimensional (3D) imaging modalities. For the intra- and extraoral 2D-imaging methods, radiographs were taken of both the left and right sides. For the 3D-radiographs, four radiographs with a field of view (FOV) of 5 × 5 cm were taken from every quadrant (posterior region).

For the intraoral 2D-radiographs, a dental X-ray tube (Heliodent®, Sirona Dental Systems GmbH, Bensheim, Germany) was used in combination with a digital sensor 3 × 4 cm at 60 kV and an exposure time of 0.08 or 0.16 s, a digital sensor at 70 kV and 0.08 s of exposure, and an Xios Scan imaging plate size 3 (27 × 54 mm) at 60 kV and 0.32 s exposure. The tube current was 7 mA.

For extraoral radiographs, the *Bitewing* (BWpan) and the not yet released *Bitewing* + (BW +) programs of the Sirona Orthophos SL-3D (Sirona Dental Systems GmbH) were utilized. For BWpan, the 2D panoramic x-ray sensor was employed at 69 kV and 12 mA (Pat 3-setting from manufacturer) as well as at 72 kV and 14 mA (Pat 4-setting from manufacturer). The same tube settings were employed for BW + , which, however, was operated using the 3D sensor of the device.

For CBCT imaging, a FOV of 5 × 5 cm was utilized for each quadrant, in combination with a high and low dose (HD and LD, respectively) setting. X-rays were taken with the Sirona Orthophos SL 3D. The details of radiographic image recording are summarized in Table 1.

**Table 1:** Exposure parameters and abbreviations used in this study

Mode	Localisation	Description	Abbreviation	kV	Exposure time
2D	Intra-oral	Digital sensor	Sensor (60 kV, 0.08 s)	60, 7 mA	0.08 s
			sensor (60 kV, 0.16 s)		0.16 s
			sensor (70 kV, 0.08 s)	70, 7 mA	0.08 s
		Imaging plate	Imaging plate	60, 7 mA	0.32 s
	Extra-oral	Panorama bitewing (2D X-ray sensor)	BWpan (Pat 3)	69, 12 mA	8.8 s (for both sides)
			BWpan (Pat 4)	72, 14 mA	8.8 s
		Bitewings plus (BW +) (3D sensor)	BW + (Pat 3)	69, 8 mA	6.5 s (for both sides)
			BW + (Pat 4)	72, 8 mA	6.5 s
3D	Extra-oral	HD (5 × 5 FOV), 80 µm voxel size	HD CBCT	85, 6 mA	14.4 s
		LD (5 × 5 FOV) 160 µm voxel size	LD CBCT	85, 10 mA	2.2 s

Intraoral radiographs were taken with the Heliodent plus® whereas extraoral radiographs were recorded using the Orthophos SL-3D

## Reference scans

Following the completion of all imaging, the posterior teeth were removed from the edentulous cadavers and scanned using a micro-computed tomography (µCT) scanner (Scanco Medical AG, Wangen-Brüttisellen, Switzerland) at 70 kVp, 114 µA, 250 ms integration time, and 10.4 µm spatial resolution. Transplanted teeth were carefully removed and also scanned using µCT employing the same parameters. On trained author (H.E.) evaluated all µCT scans. They served as a reference for the actual dimensions of the carious lesions.

## Effective dose measurements

The effective dose for each imaging combination was determined using the method by Ludlow et al. [13]. The organ doses were measured with 24 dosimeters placed at anatomically representative sites within a head phantom. An Alderson-Rando-phantom was used, and the dosimetry system was a myOSL chip (RadProInt GmbH, Remscheid, Germany) with optically stimulated luminescence dosimeters based on beryllium oxide.

The effective dose for each imaging combination was determined for all available scan options (Pat 1–Pat 4 as defined in Table 1, with and without height collimation) by performing each scan 10 times and subsequently averaging the results for higher accuracy. Height collimation was employed during conduction of the study and allowed to visualize the crowns and the upper part of the roots only. No collimation for the left or right site was employed, neither for the study nor for the effective dose measurements. Thus, the right and left site were recorded simultaneously.

## Provision of the radiographs

All radiographs were pseudonymized using a randomly selected, unique integer value. For each radiograph, a separate patient record was created in *Sidexis 4* (Sirona Dental Systems GmbH) and labeled as Patient 1, Patient 2, Patient 3, etc. [There was no correlation between the pseudonymization of cadavers and the selected scan parameters, i.e., Pat 1, Pat 2 etc.)]. All images except the BW + X-rays were provided in *Sidexis 4*, whereas for BW + , a special viewing tool, i.e., *XSensTest* (Sirona Dental Systems GmbH), was utilized, because BW + viewers had not yet been incorporated into *Sidexis 4*. The XSensTest tool allowed the adjustment of angles, scrolling through the layers, as well as contrast and gamma correction.

The device category, i.e., intraoral sensor, imaging plate, BWpan, BW + or CBCT, was discernible for the experienced observers, whereas all observers were blinded to the respective specimen.

## Calibration meeting

**Fig. 1:**
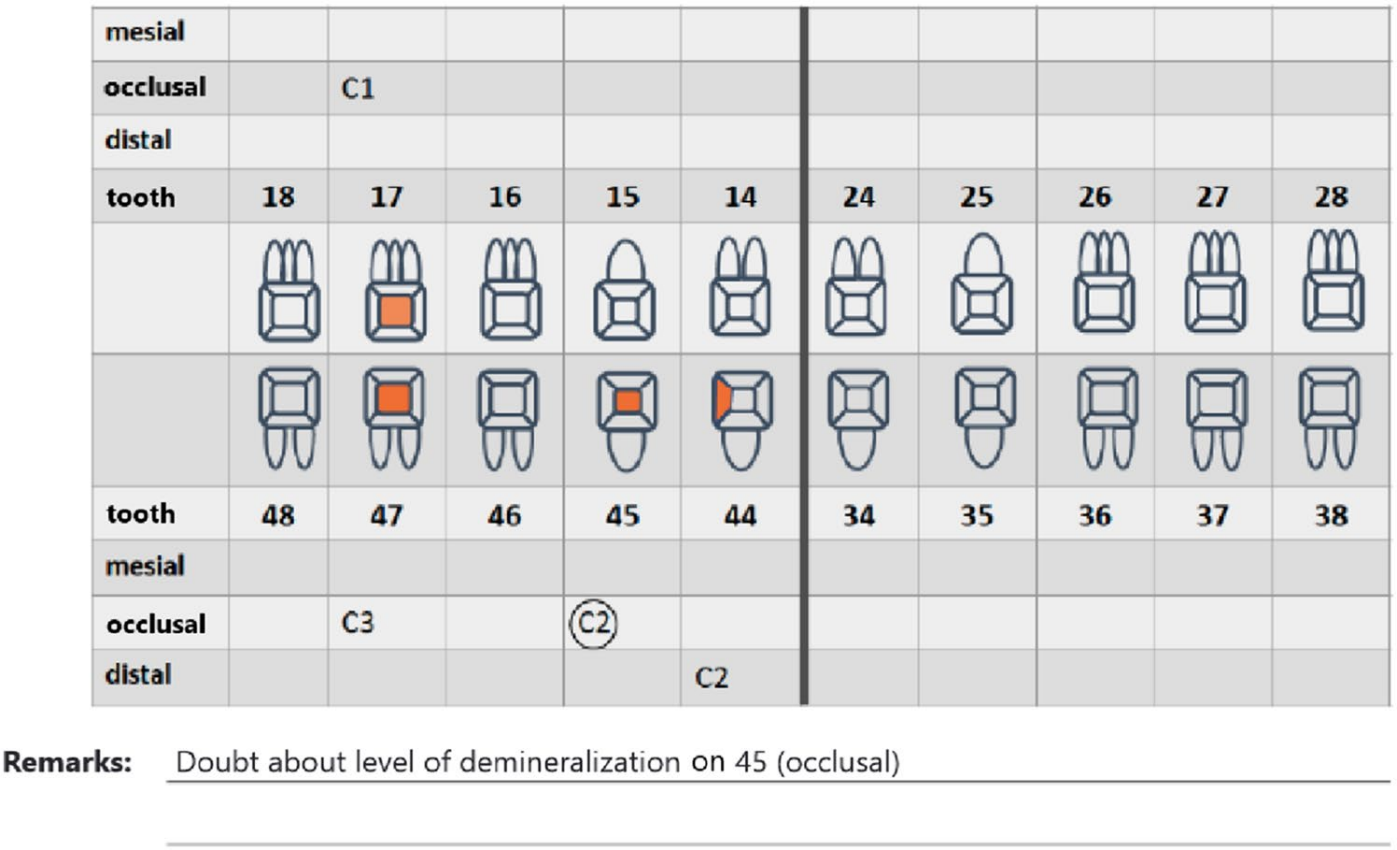
Dental charts to score the localization (marking the region) and extension of the caries lesions (specification of C1 to C4). A circle could be used in case of doubt

The observers were introduced to the novel BW + technology and the respective viewer (*XSensTest)* by means of a live demonstration. In addition, examples how to fill the score sheet were presented and discussed (Fig. 1).

## Subjective rating (pre-assessment)

Prior to the radiological assessment, the observers received a questionnaire, inquiring their subjective assessment of their personal skills in radiological caries detection, as well as their personal opinion on the suitability of BW + as well as CBCT to assess the potential benefits of 3D techniques and the need for improvements to conventional techniques.

## Radiological assessments

**Fig. 2:**
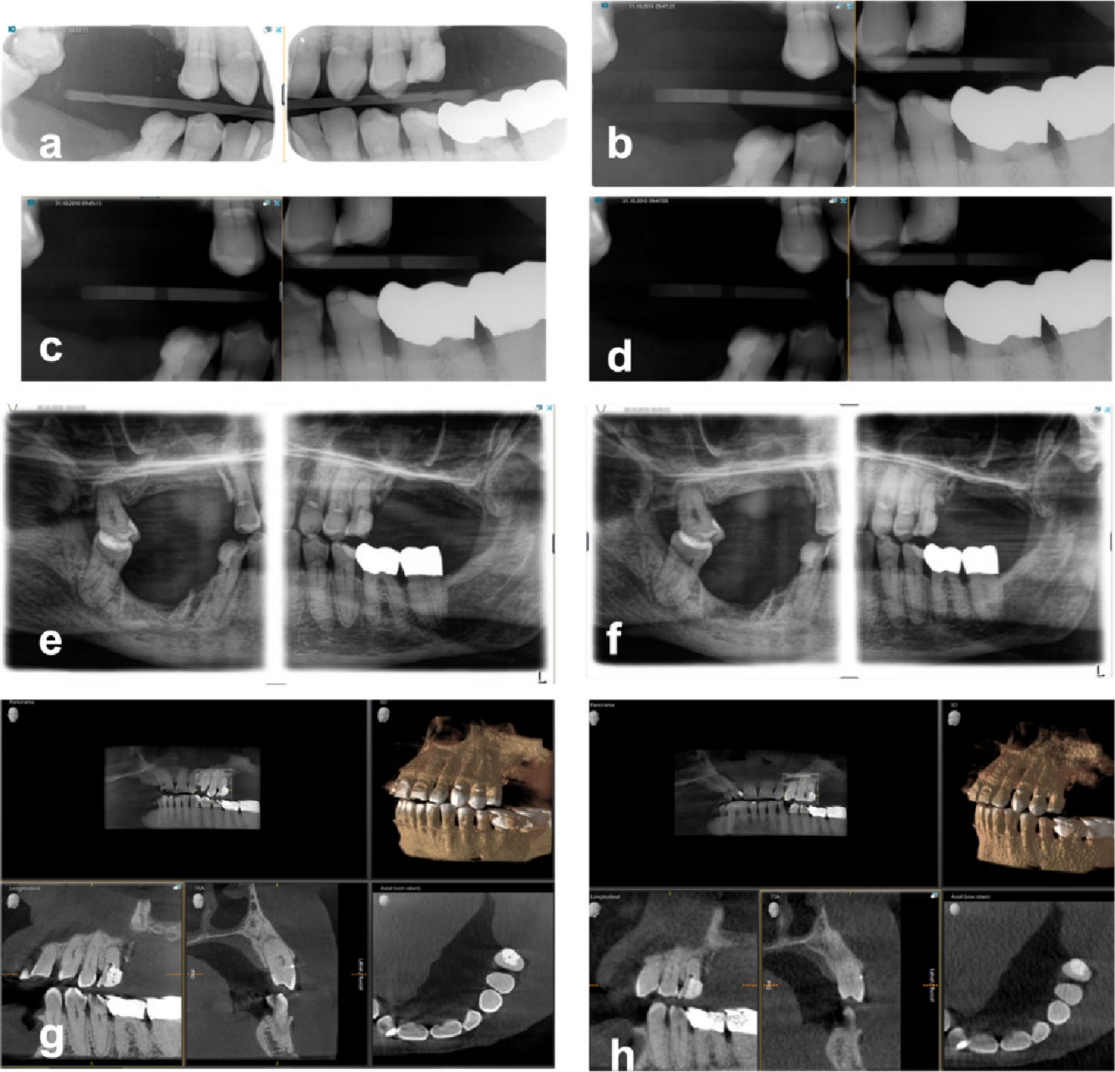
Representative examples of bitewings recorded using the (**a**) imaging plate (60 kV, 0.32 s) (**b**) digital sensor (60 kV, 0.08 s), (**c**) digital sensor (60 kV, 0.16 s), (**d**) digital sensor (70 kV 0.08 s), (**e**) BWpan (69 kV, 12 mA), (**f**) BWpan (72 kV, 14 mA), (**g**) CBCT HD (85 kV, 6 mA), (**h**) CBCT LD (85 kV, 10 mA)

For the caries diagnostics, evaluation forms showing the names of radiographs to be evaluated in randomized order were handed out. Each page contained two specifically designed dental charts (Fig. 2) that had to be completed for every radiograph. Mesial, distal und occlusal could be specified as positions for caries evaluation by marking the respective areas on the chart and entering the value in the correct row. Apart from the position of the lesion, the level of demineralization had to be determined by scoring from C1 (lesion in the outer enamel), C2 (lesion in the inner enamel), C3 (lesion in the outer dentin), to C4 (lesion in the inner dentin) or S for secondary caries. If no value was provided, it was scored as 0 (no lesion detectable). In case of hesitation regarding the correct diagnosis, observers could draw a circle around their respective entry.

On day 1, all radiographs had to be evaluated by all ten observers. 96 teeth per setting and device were examined. This corresponded to 6 × 96 = 576 teeth that had to be screened in 2D and 4 × 96 = 384 teeth that had to be evaluated with BW + and CBCT per observer, respectively. Thus, a total of 9600 teeth were scored on day 1 by all observers. This results in 28,800 possible lesions (mesial, occlusal, distal) that could be selected in the study.

On the consecutive day 2, selected radiographs were repeatedly viewed by the observers to assess intrarater reliability. Each observer, therefore, received another four images from each imaging type (BWpan, BW + , imaging plate, digital sensor and CBCT) that had been selected at random. This led to a total of 128 teeth, i.e., 384 positions for possible carious lesions per device or setting and, therefore, 3,840 positions per observer. These evaluations were then compared to the µCT reference images. µCT images were evaluated in the viewing program V6.6 (Scanco Medical AG, Wangen-Brüttisellen, Switzerland) and caries was classified into severity levels: C1–C4, secondary caries.

## Subjective rating (post-assessment)

After radiological assessments at day 2, observers were asked to complete a second questionnaire asking on to subjectively score the benefits and fields of applications of BW + compared to 2D- and other 3D-modalities in caries diagnostics (Appendix 2).

## Statistical analysis

The statistical analysis was performed using the software program R [14]. Data evaluation was performed in a two-step procedure. First, for every lesion type (C1 to C4 and secondary caries), the frequency of detecting any type of lesion at the respective position was determined for every observer. Second, the frequency of correct classification was assessed. The R package *ggplot2* [15] was used to create boxplots for descriptive purposes based on the scores obtained at day 1. Intrarater reliability was assessed using the Fleiss’ kappa measure by comparing scores from days 1 and 2. Inter-rater reliability was assessed by utilizing the different scores from day 1 from all observers. Pearson’s Chi-squared test was used to compare the different imaging modalities. Results were found significant for *P* < 0.05.

## Results

### General outcomes

**Fig. 3:**
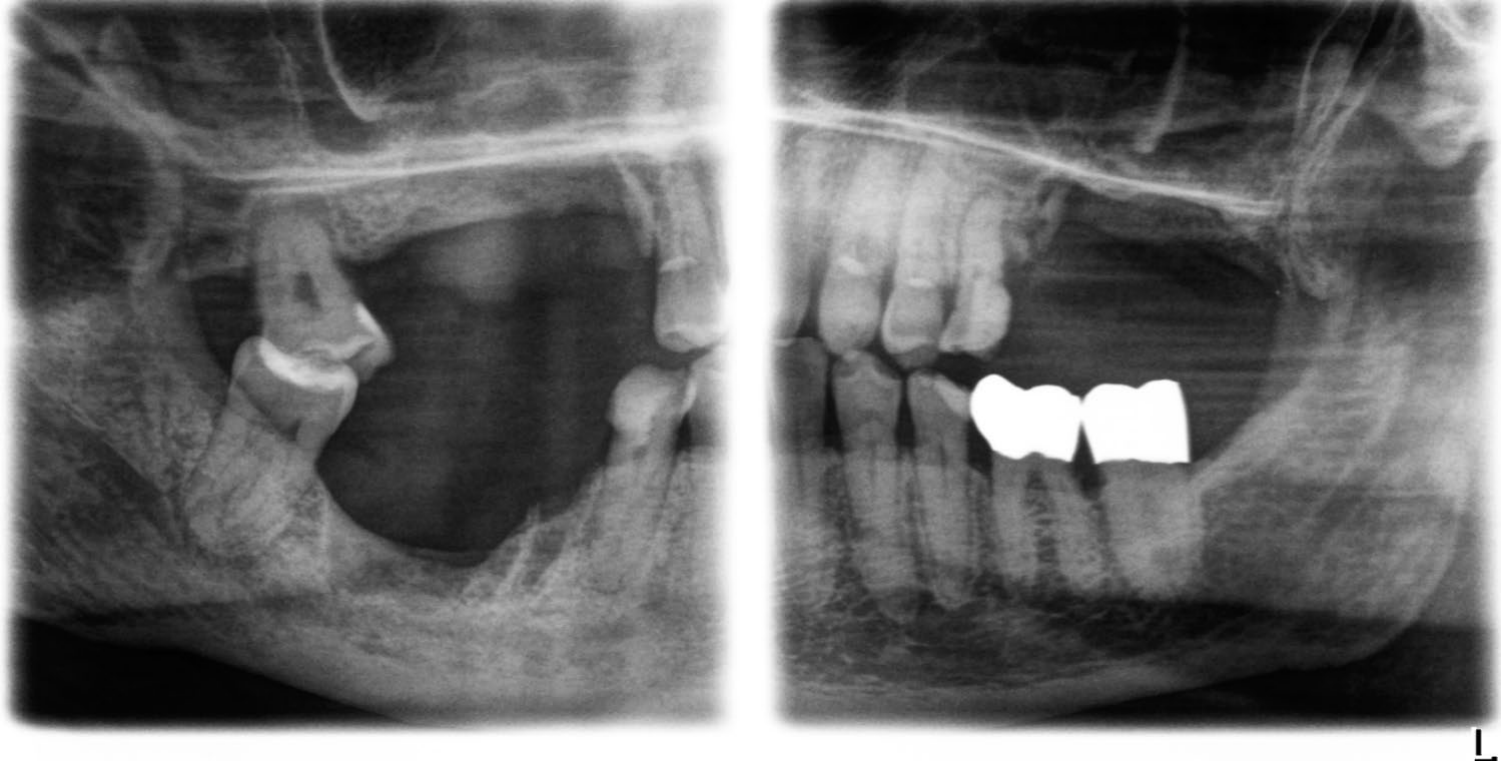
Example for a Bitewing + image shown in the XSensTest program (same cadaver and dentition as in Fig. 2)

The mean work experience of the ten observers amounted to 11 years (minimum 1.5 years, maximum 36 years). 60% of observers were specialists in oral surgery, whereas 40% were enrolled in postgraduate training. Nine from ten observers held the qualification for CBCT imaging in dentistry, while one observer was in the process of acquiring it. Recording of radiographs was completed successfully with all machines and setting combinations (Fig. 3).

## Effective dose measurements

The effective dose values of BW + scans are reported in Table 2, and were in the range 23.9–32.2 µSv, and higher doses were found with increasing tube voltage from 63 to 72 kV. By collimating the height, the effective dose was reduced by around 8 µSv regardless of the tube voltage.

**Table 2:** Determined effective doses for the scan options Pat 1–Pat 4

	Effective Dose (µSv)	Effective Dose (µSv) including height collimation
BW + (Pat 1;63 kV;8 mA)	23.9	15.7
BW + (Pat 2;66 kV;8 mA)	26.5	17.2
BW + (Pat 3;69 kV;8 mA)	28.2	21.4
BW + (Pat 4;72 kV;8 mA)	32.2	24.4

## Subjective rating (pre-assessment)

All observers responded that they consider panoramic x-rays to be the most suitable for patient screening. For caries diagnostics, intraoral bitewing radiographs with digital sensors would be their primary choice.

The majority of observers rated their experience in caries detection as high (22.2% very high, 55.6% high, 22.2% moderate) and their knowledge about caries detection as high to moderate (11.1% very high, 44.45% high, 44.45% moderate). None of the participants reported having very low or low experience and knowledge in caries diagnosis.

Prior to study participation, observers rated 3D-like imaging for caries diagnostics as useful, particularly in cases of crowding and low qualification of the staff. In this context, 33.3% found it was very useful, 44.4% useful and 22.2% were undecided.

## Radiological assessment

All observers completed day 1, whereas one observer dropped out at day 2 owing to illness. One observer did not evaluate on the second day for intrarater reliability.

**Fig. 4:**
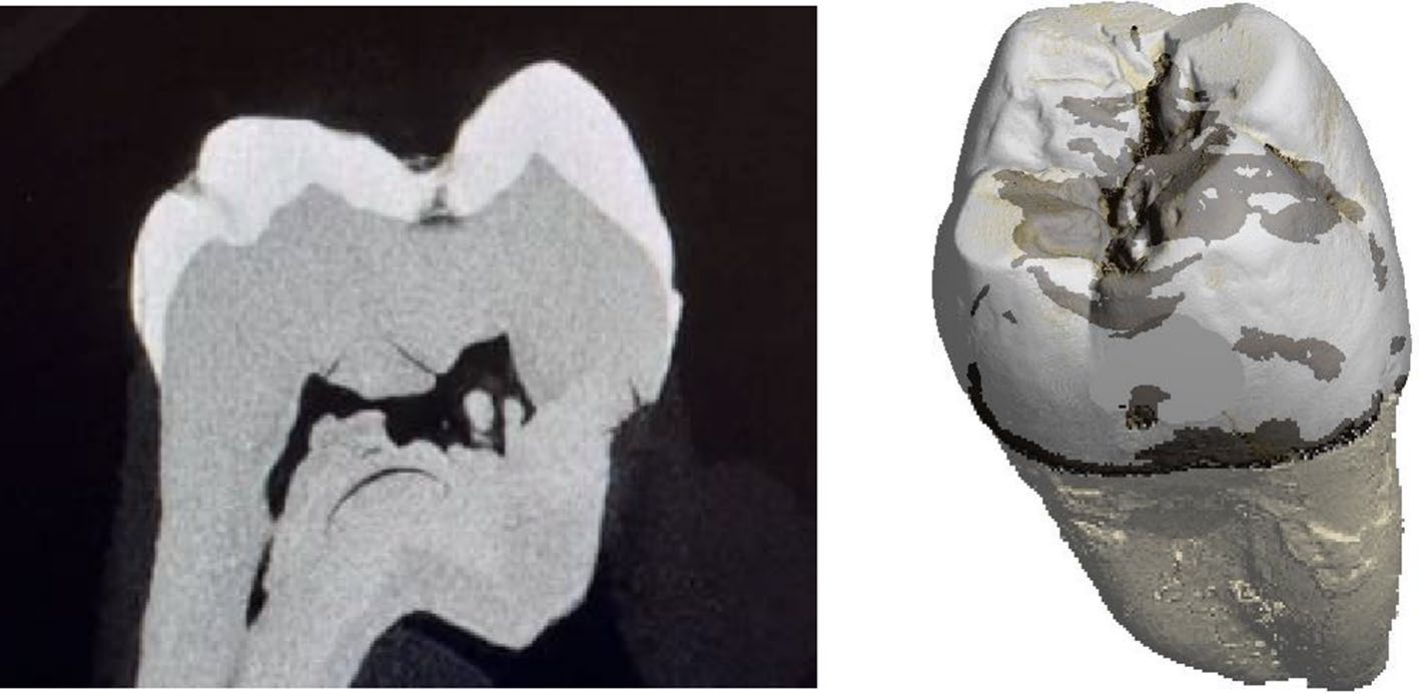
µCT image showing an occlusal C2-lesion on a molar in a 2D-slice (left), and the corresponding 3D-rendered image (right). Dark parts visualize caries. The µCT images were used as reference

When comparing the caries detection rate of the observers across all imaging methods with the caries detected through µCT evaluation (Fig. 4), differences were observed among the groups.

**Fig. 5:**
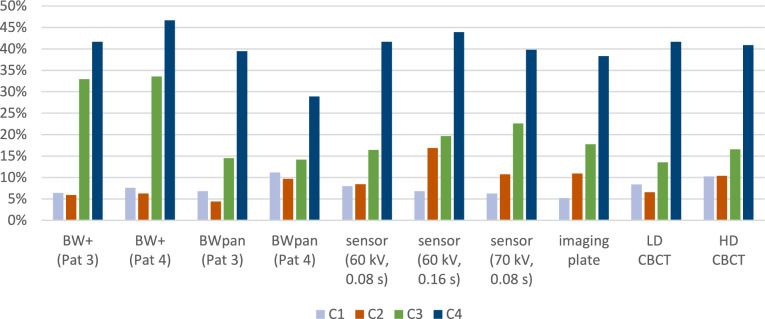
Sensitivity of the imaging modalities in relation to the degree of caries. It becomes obvious that C3 caries had the highest sensitivity with BW + followed by the sensor (70 kv, 0.08 s and 60 kv, 0.16 s). Regardless of the imaging technique, a high proportion of false negative findings are found

Out of the 35,799 possible lesions the observers evaluated, they rated 89% positions as grade 0, i.e., having no visible demineralization in the radiographs, 0% as C1, 2% as C2, 4% as C3, and 5% as C4. Contrarily, µCT scans revealed the actual number of positions with grade 0 at only 63%, C1 at 9%, C2 at 11%, C3 at 11%, and C4 at 6%. Therefore, the smallest discrepancies of 1% between the radiographic evaluation and actual demineralization were found for grade C4, i.e., caries of the inner dentin. This translates to 1793 out of 2152 positions where C4 was correctly diagnosed by the ten observers. However, less than half of C3 caries, only a fraction of C2 caries, and almost no C1 caries was detected by the observers. Overall, the observers did not detect 70% of caries, especially initial enamel lesions (Fig. 5).

## Sensitivity

**Fig. 6:**
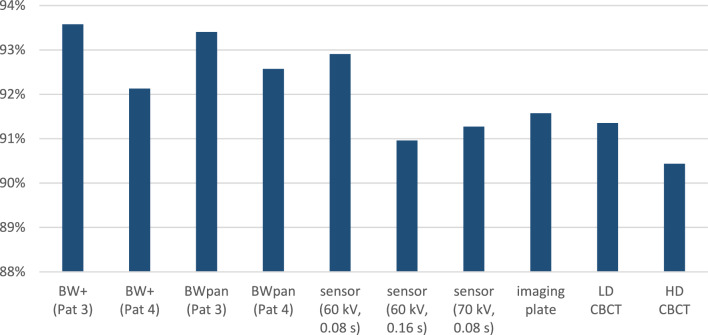
Specificity of all imaging modalities was in general high and ranged between 90,5% and 93,6%. The detailed values per lesion type (no caries, C1–C4) can be found in Table 3

Conventional extraoral BWpan (Pat 3) and BW + showed comparatively low sensitivity for C1 and C2 caries detection with a detection rate of around 12% (Fig. 6; Table 3). The highest sensitivity for C1 and C2 caries were found for the intraoral digital sensor with a voltage of 60 kV and an exposure time of 0.16 s, followed by HD CBCT and BWpan with the Pat 4-setting. BW + (Pat 3 and Pat 4) showed higher sensitivity compared to all other tested methods with up to 33.5% for C3 caries detection, and the Chi-squared test confirmed significant differences among the tested groups. For C4 caries, sensitivity was similar between all devices and settings reaching up to 47% with BW + with no statistically significant differences among groups.

**Table 3:** For C0 to C4 caries lesions, the table reports the percentage of caries detection as performed by the observers, and the percentages to which the specific lesion types (C1–C4) or no lesion was reported

	Observer assessment					Absolute amount of caries detection, regardless of depth	
Modality	No caries	C1	C2	C3	C4		
C0—no caries present							Chisq (*P* < 0.001)
BW + (Pat 3)	93.63%	0.00%	0.82%	1.87%	3.68%	6.37%	
BW + (Pat 4)	92.2%	0.22%	1.10%	2.47%	4.01%	7.80%	
BWpan (Pat 3)	93.46%	0.00%	0.33%	2.80%	3.41%	6.54%	
BWpan (Pat 4)	92.64%	0.11%	0.49%	3.13%	3.63%	7.36%	
Sensor (60 kV, 0.08 s)	92.97%	0.49%	1.70%	2.20%	2.64%	7.03%	
Sensor (60 kV, 0.16 s)	91.04%	0.71%	1.43%	3.57%	3.24%	8.96%	
Sensor (70 kV, 0.08 s)	91.35%	0.78%	1.72%	2.99%	3.16%	8.65%	
Imaging plate	91.65%	1.32%	1.21%	2.69%	3.13%	8.35%	
CBCT (LD)	91.43%	0.11%	0.71%	3.41%	4.34%	8.57%	
CBCT (HD)	90.52%	0.11%	0.94%	3.55%	4.88%	9.48%	
C1 caries present							Chisq (*P* < 0.001)
BW + (Pat 3)	93.6%	0.00%	0.40%	3.60%	2.40%	6.40%	
BW + (Pat 4)	92.4%	0.40%	1.60%	4.40%	1.20%	7.60%	
BWpan (Pat 3)	93.2%	0.00%	0.80%	3.20%	2.80%	6.80%	
BWpan (Pat 4)	88.8%	0.00%	2.00%	6.00%	3.20%	11.20%	
Sensor (60 kV, 0.08 s)	92.00%	0.40%	2.40%	2.80%	2.40%	8.00%	
Sensor (60 kV, 0.16 s)	93.20%	0.00%	0.80%	2.80%	3.20%	6.80%	
Sensor (70 kV, 0.08 s)	93.73%	0.39%	1.57%	2.35%	1.96%	6.27%	
Imaging plate	94.80%	0.00%	0.40%	2.00%	2.80%	5.20%	
CBCT (LD)	91.60%	0.40%	0.40%	4.40%	3.20%	8.40%	
CBCT (HD)	89.76%	0.39%	1.97%	4.33%	3.54%	10.24%	
C2 caries present							Chisq (*P* < 0.001)
BW + (Pat 3)	94.06%	0.63%	0.31%	2.81%	2.19%	5.94%	
BW + (Pat 4)	93.75%	0.00%	1.25%	1.88%	3.13%	6.25%	
BWpan (Pat 3)	95.63%	0.00%	0.63%	1.56%	2.19%	4.38%	
BWpan (Pat 4)	90.31%	0.00%	1.88%	5.31%	2.50%	9.69%	
Sensor (60 kV, 0.08 s)	91.56%	0.00%	3.13%	2.19%	3.13%	8.44%	
Sensor (60 kV, 0.16 s)	83.13%	1.88%	5.94%	5.31%	3.75%	16.88%	
Sensor (70 kV, 0.08 s)	89.26%	0.61%	2.76%	4.60%	2.76%	10.74%	
Imaging plate	89.06%	0.63%	3.13%	3.75%	3.44%	10.94%	
CBCT (LD)	93.44%	0.31%	1.25%	2.81%	2.19%	6.56%	
CBCT (HD)	89.60%	0.31%	2.75%	3.36%	3.98%	10.40%	
C3 caries present							Chisq (*P* < 0.001)
BW + (Pat 3)	67.10%	0.65%	7.10%	10.00%	15.16%	32.90%	
BW + (Pat 4)	66.45%	0.00%	6.77%	8.71%	18.06%	33.55%	
BWpan (Pat 3)	85.48%	0.32%	0.97%	6.77%	6.45%	14.52%	
BWpan (Pat 4)	85.81%	0.00%	0.65%	5.81%	7.74%	14.19%	
Sensor (60 kV, 0.08 s)	83.55%	0.32%	5.16%	6.13%	4.84%	16.45%	
Sensor (60 kV, 0.16 s)	80.32%	0.32%	2.90%	9.35%	7.10%	19.68%	
Sensor (70 kV, 0.08 s)	77.39%	0.64%	5.73%	8.92%	7.32%	22.61%	
Imaging plate	82.26%	0.65%	1.94%	9.35%	5.81%	17.74%	
CBCT (LD)	86.45%	0.00%	1.94%	7.74%	3.87%	13.55%	
CBCT (HD)	83.44%	0.00%	2.87%	7.64%	6.05%	16.56%	
C4 caries present							Chisq (*P* = 0.06)
BW + (Pat 3)	58.33%	0.00%	0.56%	7.22%	33.89%	41.67%	
BW + (Pat 4)	53.33%	0.00%	0.56%	6,11%	40.00%	46.67%	
BWpan (Pat 3)	60.56%	0.00%	1.11%	8.33%	30.00%	39.44%	
BWpan (Pat 4)	71.11%	0.00%	0.56%	5.00%	23.33%	28.89%	
Sensor (60 kV, 0.08 s)	58.33%	0.00%	1.11%	7.22%	33.33%	41.67%	
Sensor (60 kV, 0.16 s)	56.11%	1.11%	2.22%	7.22%	33.33%	43.89%	
Sensor (70 kV, 0.08 s)	60.22%	0.00%	1.10%	7.18%	31.49%	39.78%	
Imaging plate	61.67%	1.11%	1.11%	5.56%	30.56%	38.33%	
CBCT (LD)	58.33%	0.00%	0.00%	8.89%	32.78%	41.67%	
CBCT (HD)	59.12%	0.00%	0.55%	8.84%	31.49%	40.88%	

In addition, it reports the absolute amount of caries detection. The Chi-squared test was used to compare the different image modalities, and the respective *p* values are reported

## Specificity

Extraoral bitewing radiographs, i.e., BWpan and BW + , accounted for the highest specificity with the BW + (Pat3) and PWpan (Pat3) settings reaching more than 93%. Furthermore, BWpan (Pat4) and BW + (Pat4) and the digital sensor with 60 kV and 0.08 s showed specificity of more than 92%. The lowest specificity was measured for HD CBCT images with over 90% (Fig. 6; Table 3).

## Inter- and intrarater reliability

The inter-rater agreement was fair, with a Fleiss kappa measure of 0.308 for all raters and 0.373 for caries detection in general. The digital sensor showed the highest inter-rater reliability with 0.357, followed by BW + at 0.353 and 0.316 for setting Pat 4 and Pat 3, respectively. Intrarater reliability was good to excellent with 0.73 to 0.86 across the nine observers who did the second evaluation on the next day. Experience of the observers did not affect the accuracy of caries detection.

## Subjective rating (post-assessment)

After completing the caries scoring, 80% of the observers stated that they had preferred conventional intraoral bitewing images with a digital sensor, which corresponded to the technique that was reported by all participants to be used most commonly. Reasons were the greatest self-perceived confidence with this technique, habituality, as well as the highest perceived sharpness and resolution.

For BW + difficulties in evaluation of caries lesions in proximity to metal restorations was reported, and the observers reported self-perception of uncertainty due to inexperience. Sharpness and noise were in general were perceived likewise to the different kinds of intraoral bitewing radiographs. A loss of sharpness, however, was observed whenever the layers and planes were adjusted. Moreover, the observers mentioned that they needed more time as they had to “search for the best image”. It was also mentioned that BW + was more appropriate for caries detection compared to CBCT. Regarding BW + , higher resolution was frequently desired. Advantages of BW + were specifically seen for patients with crowding in the premolar/molar region.

## Discussion

Caries is a common disease worldwide, and bitewing radiographs are most commonly used for caries screening. Nonetheless, bitewing technique is compromised by the fact that projection radiographs are used. Therefore, the novel BW + technique was developed that operates by means of tomosynthesis and enables clinicians to scroll through different layers and angles. The goal of the present study was to compare BW + with 2D- and 3D-alternatives in terms of sensitivity/specificity, reliability, perception of observers, and effective dose.

Analysis of sensitivity revealed that BW + radiographs were associated with a significantly higher sensitivity for C3 lesions, i.e., lesions of the outer dentin. This is of clinical relevance, as C3 caries is a clear indication for invasive treatment, i.e., caries excavation followed by a restauration. Specificity was relatively high as well, especially when compared to CBCT radiographs, which, however, are not frequently used for caries detection in dental practice.

Prior to the scoring, observers expected BW + to be useful for caries detection. After assessment, they reported that they would rate BW + to be more useful than CBCT. All observers stated that they would not prefer BW + over intraoral radiographs and digital sensors, to which they were more familiar. As a reason, lack of experience with the novel technique was frequently mentioned.

A possible reason why the observers did not believe BW + to be of added diagnostic value compared to digital sensors might owe to the discrepancy between their self-rated experience, and the actual caries detection rate. In fact, compared to the µCT reference images, around 70% of caries remained undetected by the observers. It remains unclear to what extent this impacted on the answers in the questionnaire and the caries detection rates. Future studies are needed to elucidate the perception of BW + once observers got familiar with the technique.

Inter-rater reliability showed fair agreement between the observers. Interestingly, clinic experience did not seem to be of relevance in caries detection, as the two observers with the greatest accuracy in caries detection ranged from 1.5 to 36 years of working experience.

Previous studies have shown that up to 90% of carious lesions are solely diagnosed through radiological imaging, while 50% of molars of 12–20 years present visually not detectable dentinal lesions [16–18]. In this context, bitewing imaging has been shown to provide extra diagnostic value compared to visual clinical examination [19] and has repeatedly been shown to be of significant diagnostic value [20–22].

Digital radiography in particular offers a quick and inexpensive method to evaluate both teeth and bone structure alike. It has also been shown to result in less radiation exposure than conventional radiographic imaging [23–25]. However, for every application the cost–benefit ratio of potential health hazards must be considered, especially in children and women during pregnancy [26, 27].

According to the European guidelines on radiation protection in dental radiology, the effective dose of intraoral bitewing radiography amounts to approximately 1–8.3 µSv, roughly a third of dental panoramic imaging (3.85–30 µSv). This translates to a risk of fatal cancer of 0.02–0.6 per million [28]. Two bitewing radiographs, i.e., one for each half of the jaw, therefore, account for approximately 1 day’s worth of background radiation, or a continental flight. For the novel BW + mode and the tested Pat 3/Pat 4 settings, doses were slightly higher than for conventional intraoral radiographs, which amounted to 15 µSv for a full intraoral examination [29]. According to the dose measurements specified in the handbook of the CBCT, effective dose values of BW + were slightly higher than the CBCT LD images (28.2 µSv compared to 4 µSv, Pat 3 setting), but lower compared to CBCT HD images (28.2 µSv compared to 69 µSv maxillary / 71 µSv mandibular, Pat 3 setting). It has to be noted that the values for CBCT are much lower compared to literature, and that no information is given in which region of the jaw they were reported. These higher values can be justified by the benefits of a local third dimension. Nevertheless, the European guidelines recommend no more than six-monthly intervals for posterior bitewing radiographs for high-risk, and annually for patients with moderate caries risk.

Furthermore, European guidelines, as to which intervals are adjusted for low-, moderate-, and high-risk patients, may differ from other international guidelines, as a review by Goodwin et al. indicated [30]. Therefore, careful consideration of individual age, risk, added diagnostic value, and likeliness of treatment alteration through radiography must be taken to justify each decision. As caries takes up to 4 years or longer to invade the dentin through the enamel layer, annual or even biannual radiographic monitoring may not be of additional diagnostic value in case that only initial C1 lesions are present [31, 32], and is, therefore, not indicated for these cohorts of patients.

Even though projection errors occur rarely in bitewing radiography [33], they can impair sensitivity and specificity. However, it must be mentioned that there are other factors impairing bitewing radiographs, such as burn-out artefacts, and whether they are also present in BW + was not investigated in the present study.

In dentistry, cone beam computer tomography (CBCT) has become gold standard for 3D imaging. It has significantly lower radiation exposure when compared to traditional computed tomography [34] and is usually very fast in regard to recording time and image reconstruction. Despite its broad range of indications, it is commonly used in addition to previous 2D-imaging in case that further information is required. Moreover, it requires the practicing dentist to obtain an extra qualification for CBCT in many countries.

Interestingly, the inter-rater reliability was only fair in the present study. Also, the intrarater reliability varied among observers, and only two doctors reached an agreement of more than 85% between day 2 and day 1. This emphasizes that many lesions were not seen by all the observers, as reported previously by another group [35]. Hence, specific trainings for dentists to improve skills in caries detection might be beneficial, as suggested in the field of radiation protection in the EURATOM guidelines [36]. In addition, combination of human and artificial intelligence might improve detection rates in the future [37].

This design of the present study was chosen to simulate clinical reality as much as possible, without the need of exposing patients to hazardous radiation. Nevertheless, a limitation of this study is its ex vivo nature. As only two specimens were in possession of their natural dentition, teeth had to be surgically transplanted into the alveolar bone of three edentulous cadavers. As this procedure was extensive and complex, the respective pre-drilled holes were used for various insertions of tooth combinations. In addition, the high number of carious lesions may not reflect a typical individual, and might have contributed to lower detection rates, as lesions might have been easier overseen by the raters. For imaging plates, it has to be noted that recording was impaired by the low intraoral temperature of the cooled cadavers. Another limitation owes to the fact that not all potential voltage and current combinations could be evaluated. Around a decade ago, a study by Hellén-Halme and Nilsson has shown that a voltage of 70 kV leads to 40–50% higher absorbed dose in patients than 60 kV [38], and another study found that a voltage of 70 kV does not result in a significantly higher sensitivity than 60 kV [39]. This is in line with the sensor recordings of the present study, even though a slight reduction in specificity was found for 60 kV. Regarding the extraoral bite wings, only the most typical dose settings (Pat 3 and Pat 4) were evaluated. Therefore, future studies are needed to evaluate the applicability lower voltage protocols, specifically for extraoral BW and BW + .

It has to be noted that the present study was designed to evaluate whether there is a general benefit of BW + . However, no focus was put on more specific fields of indication, such as its advantages for caries diagnosis in disabled patients, or in patients with strong parapharyngeal reflex. From clinical practice it is known that intraoral radiology techniques are sometimes not applicable in these patients, so extraoral approaches might become a valuable alternative here. Another field in which BW + might be beneficial was mentioned in the questionnaire, where advantages of BW + were seen for patients with posterior crowding. Indeed, clinical reality sometimes requires separate intraoral radiographs for each interproximal contact, so the overall radiation dose would be lower with the BW + technology. Another limitation of the current study is that no differentiation was performed between interproximal and occlusal caries lesions. However, in future studies, it would be interesting to assess the specific benefits of BW + at the different localizations. Furthermore, it has to be noted that calibration only included a group discussion which lesions visible on exemplary radiographs would be represented by the different scores. Despite, additional test was run to validate consistent scoring among observers. However, since all observers were part of the same department and used the scores in clinical routine, a pre-calibration was assumed.

In conclusion, and within the limitations of the present study, BW + displayed increased sensitivity for detection of C3 caries lesions, and slightly higher specificity compared to other 2D- and 3D-imaging methods. These results prove that BW + is suitable for caries diagnostics and also has the potential to become a relevant tool for intraoral bitewing diagnostics in the future. Despite the diagnostic advantages, the risk–benefit ratio should be weighed up individually for each patient according to age and caries risk, as the 3D effect results in increased radiation exposure.

## Data Availability

Data will be provided upon reasonable request.
